# An emigration versus a globalization perspective of the Lebanese physician workforce: a qualitative study

**DOI:** 10.1186/1472-6963-12-135

**Published:** 2012-05-30

**Authors:** Elie A Akl, Nancy Maroun, Aline Rahbany, Amy Hagopian

**Affiliations:** 1 Department of Medicine, State University of New York at Buffalo, Buffalo, NY, USA; 2 Department of Family Medicine, State University of New York at Buffalo, Buffalo, NY, USA; 3 Department of Clinical Epidemiology and Biostatistics, McMaster University, Hamilton, Canada; 4 Department of Sociology, Buffalo State College, Buffalo, NY, USA; 5 Independent researcher, Beirut, Lebanon; 6 School of Public Health, University of Washington, Seattle, WA, USA; 7 Department of Medicine, State University of New York at Buffalo, ECMC-CC 142, 462 Grider St., Buffalo, NY, 14215, USA

**Keywords:** Emigration and immigration, Health manpower, Medical education, Physicians

## Abstract

**Background:**

Lebanon is witnessing an increased emigration of physicians. The objective of this study was to understand the perceptions of Lebanese policymakers of this emigration, and elicit their proposals for future policies and strategies to deal with this emigration.

**Methods:**

We conducted semi-structured individual interviews with the deans of Lebanon’s seven medical schools, the presidents of the two physicians professional associations, and governmental officials. We analyzed the results qualitatively.

**Results:**

Participants differed in the assessment of the extent and gravity of emigration. Lebanon has a surplus of physicians, driven largely by the over-production of graduates by a growing number of medical schools. Participants cited advantages and disadvantages of the emigration on the personal, financial, medical education system, healthcare system, and national levels. Proposed strategies included limiting the number of students entering medical schools, creating job opportunities for graduating students, and implementing quality standards. Most participants acknowledged the globalization of the Lebanese physician workforce, including exchanges with the Gulf region, exchanges with developed countries, and the involvement of North American medical education institutions in the region.

**Conclusion:**

Many Lebanese policy makers, particularly deans of medical schools, perceive the emigration of the physician workforce as an opportunity in the context of the globalization of the profession.

## Background

Lebanon has witnessed a rapid expansion of its medical education capacity with the establishment of three new private medical schools since 2000 for a total of seven schools (Additional file 1). The two oldest medical schools were established respectively in 1868 and 1883. While no formal accreditation system of medical schools currently exists, there are ongoing efforts to create one. All schools are university-based and these universities make all academic decisions connected to the degree course. The demand for physicians from higher-income countries, especially Gulf countries, the U.K., Canada, the U.S. and Australia, has likely fuelled the growth in private medical schools in many low-income countries, including Lebanon.

The substantially increased numbers of medical school graduates in Lebanon might have contributed to the significant increase in physician emigration reported in the mid 2000’s [1]. In 2004, about 40% of those who graduated from Lebanese medical schools in previous 25 years were active physicians in the US [2]. A recent survey of students graduating from Lebanese medical schools found 96% of respondents intended to work abroad, [3] in large part because of a perceived oversaturation of the local physician job market [4]. While no study has formally documented such oversaturation or assessed the distribution of Lebanese physicians, in 2007 there were 10,918 registered physicians (physician density of 2.73 per 1000) [5]. In 2009, 72% of Lebanese physicians reported practicing primarily in urban settings [5]. Other developing countries have also witnessed a worsening migration of their physician to developed countries [1].

The increase in Lebanon’s physician supply capacity may be beneficial in a number of ways, however. A migrant professional workforce can have positive effects on the national economy through remittances sent back to family members, [6] and by spurring more people to go to medical school than might have in the setting of no migration opportunity. When physicians return home, they can transfer new expertise to the existing workforce [7,8]. In the specific case of Lebanon, there have even been efforts to establish the country as a regional ‘academic hub,’ a proposal that would benefit from increased physician capacity [9].

Although potentially important, Lebanon has not adopted a national medical workforce plan, perhaps because the process of establishing one would be contentious. The objective of this study was to understand the perceptions of Lebanese policymakers on the emigration of Lebanese medical graduates, and elicit their proposals for future policies and strategies to deal with this emigration.

## Methods

### Setting

Lebanon is a middle-income country on the Eastern Mediterranean Coast with a recent history of civil instability. There are seven medical schools in Lebanon—one public and six private— with three private schools established in the last decade (Additional file 1). Physicians who are not graduates of the Lebanese University have to pass an oral test called the ‘colloquium exam’ in order to obtain a license to practice medicine in the country. Licensed physicians are members of one of two associations called the Lebanese Orders of Physicians, depending on the geographical location of their practice. More than 70% of physicians are in solo practices, primarily in urban settings [5].

### Participants

We used purposive sampling to recruit the deans of medical schools, the presidents of the two Lebanese orders of physicians, and governmental officials. Two of the investigators (EAA, NM) established the contact with potential participants. The Institutional Review Board of the University at Buffalo approved the study and all participants provided informed consent.

### Data collection

We chose to conduct semi-structured individual interviews to collect the experiences, perceptions, and practices of participants. In the summer of 2008, two of the investigators (EAA, NM) used the following standard set of guiding questions in the participant’s preferred language (Arabic, English, French):

· How do you perceive the extent of the emigration of Lebanese medical graduates?

· What are the causes of this emigration?

· What has been the contribution of the medical school to this emigration?

· What are the advantages of this emigration?

· What are the disadvantages of this emigration?

· What should be the role of the medical education system in Lebanon, as it relates to the emigration of Lebanese medical graduates?

· What are the policies needed to accomplish this role?

### Data analysis

We transcribed verbatim the audiotaped discussions and translated them to English. One investigator (AR) manually coded the text with labels encapsulating the substantive themes inherent in the discussions. She then analyzed the themes and clustered them into concepts. A second investigator (EAA) reviewed those results in light of the raw data. We used deductive analysis to evaluate themes on the emigration of Lebanese physicians (e.g. causes, advantages, disadvantages). We analyzed the data for emerging concepts using an inductive analysis processes [10].

## Results

The informants in this study included the deans of the seven Lebanese medical schools, the presidents of the two Lebanese orders of physicians, the director general of the ministry of higher education, and a member of the parliamentary committee on higher education. Only one potential participant (parliament member) refused to participate with providing a reason.

Our deductive analysis generated themes on the emigration of Lebanese physicians (including its extent, nature, causes, advantages, disadvantages, and potential solutions). The inductive analysis generated findings relating to the theme of globalization of the Lebanese physicians workforce. We detail these results separately in the following sections.

### Emigration of Lebanese physicians

#### Extent and nature

Deans of faculties, presidents of the Orders and the official provided varying estimates of the magnitude of emigration of new Lebanese medical graduates, with guesses ranging from 25% and 50%. Participants complained of the lack of accurate data, blaming it on governmental agencies and the Orders of Physicians.

The majority of the deans and the Presidents of the Orders differentiated between temporary and extended migration. Temporary migration occurs for specialization (post graduate training) and/or for getting abroad experience for a limited period of time. According to one of the participants, ‘you cannot really call the temporary migration as emigration; those are doctors who go to work in the Arabic Gulf region for example, come to Lebanon 3 or 4 times a year to see their families. Doctors who seek extended emigration ‘are those who don’t have any thoughts of coming back,’ described by one dean as the ‘ultimate emigrants.’

#### Motivations

The two most frequently cited reasons to emigrate were the challenging socio-economic conditions and the unstable security situation in Lebanon: ‘People want to work and live peacefully,’ one participant told us.

A frequently discussed push factor was the surplus of physicians: ‘This is all because of the surplus of physicians in Lebanon - we have 12,000 when we need a maximum of 5,000,’ said one dean. The surplus reduces job opportunities and makes practice conditions more financially and professionally challenging. Participants agreed the lack of national planning is worsening the situation.

Presidents of the two Orders of physicians and the government official argued that the abundance of medical schools was a major reason for the surplus of physicians in the country. The shortage of residency training programs also motivates students to emigrate for their postgraduate training. Post-graduate training has no public subsidy. Two respondents note that Lebanese young people have always sought better quality training and job opportunities abroad, regardless of their field of education.

The deans of the medical schools contended the number of medical schools was not the driver of the surplus, and that students interested in medical studies would pursue their aspirations even if they had to study abroad. Indeed, the deans pointed to Lebanese students completing medical studies in Eastern Europe, and returning to work in Lebanon.

Participants claimed people have a right to education in the area of their choice. One dean said, ‘You cannot stop people from doing what they want to do; especially in Lebanon; that's why you have 20 newspapers, 14 TV stations and everything that we don’t need; we have them in excess.’

Finally, participants cited the limited job opportunities in the region as a cause of emigration.

#### Advantages

Participants discussed advantages of the emigration of physicians on the personal, medical education system, healthcare system, and national levels.

On the personal level, most participants argued that emigration offers Lebanese physicians higher earnings, better working conditions, and advanced career options. One of the deans said, ‘In a country where you have an excess of physicians, through emigration they can all find jobs; whether in research, in teaching or in symposiums.’

The deans of medical schools said the requirement of their graduates to compete in the ‘global market’ (e.g., for residency positions) creates an incentive to set high standards for their educational programs. One dean told us that in spite of the lack of unified regulations in the country, Lebanese schools are able to ‘benchmark with the international compass’ of medical schools by monitoring the passing rate on international exams.

On the level of the healthcare system, participants said repatriated physicians share their advanced training with peers when they return home, through teaching and research. In addition, patients benefit directly from the knowledge and skills physicians acquire abroad when they relocate to Lebanon, or even when they provide clinical services during short visits.

On the national level, the main benefit named was the positive effect on the economy. One of the deans said, ‘The deficits of the Lebanese economy have been compensated by remittances from abroad.’ An additional benefit was the positive effect these migrants can have on the image of the country: ‘we can benefit from these people, these are our ambassadors.’ They also serve as a social network resource for Lebanese seeking advanced medical care abroad.

#### Disadvantages

Participants discussed disadvantages of the emigration of physicians on the personal, healthcare system, and financial levels. On the personal level, some participants expressed concerns that emigration creates family separation and weakens family bonds in general.

On the financial level, participants considered the emigration of physicians as a financial loss for both their families and the country. According to the one of the participants, the education of a doctor costs around 120,000 USD: ‘we are putting efforts and money on young people's education and they are helping others and not us.’

On the level of the healthcare system, a number of participants were concerned that the ‘elite’ or ‘the best graduates’ are the one emigrating since it is ‘easier for them to be accepted abroad and to settle.’ As a consequence, and according to one of the deans, ‘what is left in the Lebanese market are students that are average or below average, less quality,’ which could negatively affect the quality of the healthcare. Another participant said, ‘in the past, physicians, the best of them used to specialize and then come back to Lebanon. While now the best specialized do not want to come back here. And this situation will have reflections on the quality of the medical care on the long term.’

Furthermore, two participants were concerned about the quality of Lebanese physicians graduating from medical institutions abroad, specifically Eastern Europe. One dean said, ‘wouldn’t be annoying if there were standards all would follow,’ ironically referring to the lack of a strict licensing processes for those entering the workforce. This could ‘negatively affect the quality of the profession since not all of those who emigrate access high quality knowledge and training.’

#### Potential solutions

Solutions proposed by our subjects included limiting the number of students entering medical schools, limiting the number of students seeking medical education, creating job opportunities for graduating students, and implementing quality standards.

A number of participants raised the need to limit the number of physicians entering the job market by limiting the number of students entering medical schools. The presidents of the two Orders of physicians and the government official discussed how Lebanon could establish public policy to determine the number of physicians needed in the country (he called it ‘numerus clausus’). ‘If we need 300 physicians per year,’ he said, ‘then the three hundred who pass the unified program's exam get to go to any medical school in Lebanon they like. Even the ones that will go to study medicine abroad should take the exam before they leave.’

The presidents of the Orders also suggested discouraging those who seek medical degrees with false expectations of achieving wealth by offering an orientation to high school students. Similarly, when medical doctors seek specialization, a Lebanese government office could steer doctors towards the specializations for which there is economic demand.

Participants also discussed how to provide jobs for new medical graduates. One dean said, ‘the whole political and social environment should be conducive for these people to remain in the country to conduct research or do other professional work.’

Participants agreed that reducing the number of medical schools in Lebanon would be difficult given the political nature of the decision. ‘There is no way, politically speaking, to close any university. That’s why it would be better to improve its level. If we are able to improve its level through an objective system, that has nothing to do with the political situation, and which is the accreditation system, then we would be assuring an acceptable level.’

Repeatedly in the discussions, study participants highlighted the importance of implementing quality standards, through accreditation of medical education institutions, and certification of medical graduates through licensure and board exams. These evaluations of new graduates would replace the current ‘colloquium’ test perceived as biased and ineffective. The deans also discussed the value of standardized testing to monitor the quality of entering medical students.

One of the deans argued that the accreditation of medical schools and the administration of board exams should be conducted by a non-Lebanese entity, e.g., from the United States, Western Europe, or Australia. These entities have the experience, they argued, but also they could ensure independence ‘because we know what are the political implications,’ suggesting that local entities are may not be independent and unbiased.

The deans claimed the lack of follow-up on accreditation from the Orders of physicians and the lack of collaboration within government and between schools were major barriers. A president of one of the Orders stated ‘everything is taking place randomly.’ The other president said, ‘the problem in Lebanon is not the lack of ideas, but rather the lack of will to make things better and someone to make a decision.’ The government official said the delay in accreditation progress is owing to the fact that ‘90% of the universities are private, each having its own affiliation with some university abroad or a certain religious community, there is no control of the State on the number and qualifications of medical students enrolled each year in these schools.’

### Globalization of the Lebanese physician workforce

**Figure 1:**
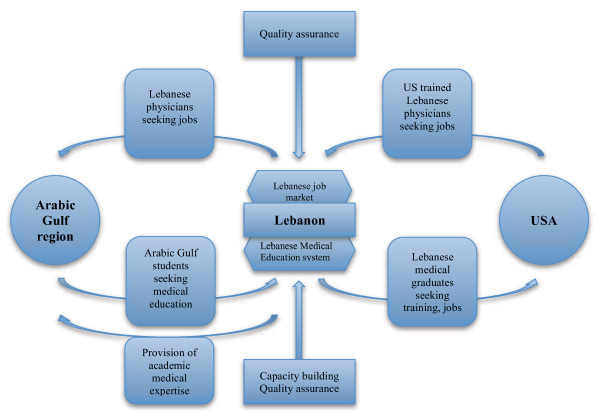
Globalization of the Lebanese physician workforce.

Our participants discussed a variety of topics relating to the globalization of the physician workforce, including exchanges with developed countries, regionalization of training, cross-border quality assurance, and the involvement of North American Institutions in the Arabic Gulf region (including Weill Cornell Medical College in Qatar, Harvard Medical School Dubai Center, Johns Hopkins Medicine International in the United Arab Emirates). While exchange with developed countries relates mainly to the U.S., regionalization refers to the Arabic Gulf region. Figure 1 depicts how the concepts relate to each other.

#### Exchange with developed countries

This exchange is bidirectional. In the first direction, Lebanese medical graduates move from Lebanon to developed countries seeking post-graduate training and subsequently jobs. Presidents of the Orders of physicians and deans of faculties, alike, compared medical education in Lebanon to ‘producing' or 'manufacturing’ physicians almost as an export commodity. One dean stated: ‘we take the best students and produce the best physicians and those who don’t have a job in Lebanon will work in America or in the Gulf.’ Also, Lebanese medical graduates abroad typically help new Lebanese graduates to enroll in training programs in the institutions where they work. In the second direction, Lebanese physicians who have trained and worked in developed countries return to Lebanon seeking jobs.

#### Regionalization

This concept also consists of two directions. Lebanese physicians move to the Arabic Gulf region for jobs. One dean suggested Lebanese medical schools could be appealing to students from the Arabic Gulf countries given shortages in both physicians and medical education capacity in those countries. He argued attracting the best students from the region would improve the quality of the medical school, while acknowledging the number of Lebanese nationals who graduate may be reduced. However, he pointed to a number of barriers to this scheme: the security situation, the elevated cost (e.g., tuition, living expenses) and the lack of high quality residency training in Lebanon.

#### North American institutions and the gulf region

A number of deans raised the issue of the involvement of North American medical institutions (e.g., Weill Cornell, Harvard International, Johns Hopkins Medicine International) in the Arabic Gulf region. One dean described these institutions as ‘vultures,’ referring to their high fees for helping the Arabic Gulf establish medical education institutions and hospitals.

One dean noted his institution suggested a triangular collaboration among the Arabic Gulf institutions and the North American institutions, but the latter refused. He said that Lebanese medical institutions could provide academic medical expertise, including strategic planning, developing curricula, and training.

Another dean noted that the North American institutions are struggling with recruiting academicians to staff their regional branches. He suggested: ‘[we should] bring the physicians from abroad and place them in a medical/academic institution in Lebanon with an organized system and a chance to teach, to do research, to continue their academic careers… plus access to clinical practice in Lebanon and in the Gulf.’

## Discussion

Participants differed in their assessments of the extent and gravity of emigration. While they agreed the surplus of physicians is a main reason for migration, they disagreed on whether the number of medical schools is contributing to this surplus. Some proposed solutions, included limiting the number of students entering medical schools, limiting the number of students seeking medical education, creating job opportunities for graduating students, and implementing quality standards. An emerging theme was the globalization of the Lebanese physician workforce and the bidirectional exchange with developed countries and within the Arabic Gulf region.

This study has a number of strengths. This is the first qualitative study of Lebanese policymakers seeking to understand the emigration of physicians and identify potential solutions. In fact, we have not identified and are not aware of any similar study in other countries. Another strength is our use of standard methods in qualitative data collection and analysis. One limitation of the study is the relatively small number of participants. However, the number was determined by the scope of the study; in fact, we were able to recruit representative of all important stakeholders.

Two issues emerged from the discussion as problematic, but participants did not address them when proposing solutions. The first issue is the lack of a national plan for human resource for health. Indeed, any policies or strategies to deal with medical education and physician migration need to be part of a larger and comprehensive human resource for health plan [11,12]. Policy makers can use a number of tools specifically develop for such planning [13].

The second problematic issue is the concern that the best graduates leave. Although we are not aware of evidence to support this concern, it is consistent with concerns [14] and observations [15] reported in other countries. The repatriation of these high quality graduates, while not without challenges [16], is important in the setting of ambitions visions for the Lebanese physician workforce. The concept of the ‘academic hub’ builds on the potential to tap into a globalized and high quality Lebanese physician workforce [9] to address the serious shortage of clinicians and academicians in the neighboring Arabian Gulf countries [17].

## Conclusion

Lebanese stakeholders and policy makers seem poised to collaborate on reaching a vision for the Lebanese physician workforce. One of the top priorities would be to work together on accreditation and certification. Another priority would be to address the problem of the oversaturation of the physician job market. Academic medical institutions have only toyed so far with the idea of promoting Lebanon as an academic medical hub for the region—the time is ripe to pursue the feasibility of this notion.

Future research should track the location, expertise and career plans of Lebanese medical graduates. This is important not only for the developing plans for the Lebanese job market but also for executing some of the regional plans suggested by the deans of the medical schools.

## Competing interests

None of the authors has any conflict of interest to declare.

## Authors’ contributions

EAA contributed to study concept and design, data collection, data analysis, and drafting of the manuscript. NM contributed to study concept and design, and data collection. AR contributed to data analysis. AH contributed to study design. All authors read and approved the final manuscript.

## Pre-publication history

The pre-publication history for this paper can be accessed here:

http://www.biomedcentral.com/1472-6963/12/135/prepub

## Supplementary Material

Additional file 1 Description of Lebanese medical schools. Click here for file
