# Supplementary material for: An emigration versus a globalization perspective of the Lebanese physician workforce: a qualitative study
Source: BMC Health Serv Res. 2012 May 30;12:135. doi: 10.1186/1472-6963-12-135 (PMC3414743; doi:10.1186/1472-6963-12-135)
Supplement: Additional file 1 — Description of Lebanese medical schools. [file 1472-6963-12-135-S1.doc]

**Additional file 1:** Description of Lebanese medical schools

|  | **Instruction began** | **Admission requirement** | **Language(s) of instruction** | **Length of studies** | **Degree granted** | **Type** | **Public funds** | **Tuition** |
| --- | --- | --- | --- | --- | --- | --- | --- | --- |
| American University of Beirut (AUB) | 1868 | Bachelor degree | English | 4 years | Doctor of Medicine (M.D.) | Private | None | Not free |
| Université Saint Joseph (USJ) | 1883 | High school graduation | French | 7 years | Doctor of Medicine (M.D.) | Private | None | Not free |
| Lebanese University (LU) | 1983 | High school graduation | English, French | 7 years | Doctor of Medicine (M.D.) | Public | Publicly funded | Free |
| Beirut Arab University (BAU) | 1995 | High school graduation | English | 6 years | Bachelor degree in Medicine and Surgery (M.B.B.Ch.) | Private | None | Not free |
| University of Balamand (UOB) | 2000 | Bachelor degree | English | 4 years | Doctor of Medicine (M.D.) | Private | None | Not free |
| Holy Spirit University of Kaslik (USEK) | 2002 | High school graduation | English, French | 7 years | Doctor of Medicine (M.D.) | Private | None | Not free |
| [Lebanese American University](http://www.lau.edu.lb/) (LAU) | 2009 | Bachelor degree | English | 4 years | Doctor of Medicine (M.D.) | Private | None | Not free |
